# MiR-155 contributes to intestinal barrier dysfunction in DSS-induced mice colitis via targeting HIF-1α/TFF-3 axis

**DOI:** 10.18632/aging.103555

**Published:** 2020-07-26

**Authors:** Yujin Liu, Feng Zhu, Huarong Li, Heng Fan, Hui Wu, Yalan Dong, Si Chu, Chen Tan, Quansheng Wang, Hongxia He, Fei Gao, Xueyuan Leng, Qiaoli Zhou, Xiwen Zhu

**Affiliations:** 1 Department of Integrated Traditional Chinese and Western Medicine, Union Hospital, Tongji Medical College, Huazhong University of Science and Technology, Wuhan 430022, China

**Keywords:** miR-155, inflammatory bowel disease (IBD), intestinal barrier dysfunction, HIF-1α, TFF-3

## Abstract

Intestinal barrier dysfunction is a hallmark of inflammatory bowel disease (IBD). MiR-155 is increased in colitis and downregulates expression of hypoxia-inducible factor 1α (HIF-1α). Here, we investigated the effects of miR-155 on intestinal barrier dysfunction in dextran sulfate sodium (DSS)-induced colitis. We found that miR-155 antagomir treatment relieved weight loss and intestinal damage in IBD mouse models (P < 0.05). Furthermore, electron microscopy and immunofluorescence imaging showed that miR-155 increased intestinal barrier dysfunction and downregulated the expression of tight junction proteins in DSS-induced colitis. FG-4497, which upregulates HIF-1α expression, elicited protective effects on the intestinal barrier in DSS-induced colitis. Dual luciferase reporter assays also confirmed that miR-155 downregulated expression of HIF-1α. Finally, we discovered that HIF-1α levels were elevated by miR-155 antagomir treatment (P < 0.05) and that TFF-3 expression correlated positively with HIF-1α expression. These results suggest that miR-155 contributes to DSS-induced colitis by promoting intestinal barrier dysfunction and inhibiting the HIF-1α/TFF-3 axis.

## INTRODUCTION

Inflammatory bowel disease(IBD), a pathological state characterized by relapsing and remitting gastrointestinal (GI) tract mucosal inflammation, consists mainly of ulcerative colitis (UC) and Crohn’s disease (CD) [1]. Although the pathogenesis of IBD is still not completely understood, prior studies suggest that an abnormal activation of the mucosal immune response in genetically-susceptible individuals contributes to IBD [1]. As the initiating event of IBD, the impairment of intestinal epithelial cells that exert intestinal barrier functions plays a pivotal role in the pathophysiology of IBD and worsens as the disease progresses [2].

Numerous miRNAs regulate junction protein gene expression and maintain junction structural integrity [3, 4]. Furthermore, miR-155 promotes endothelial junction function and epithelial TJ expression in atopic dermatitis [3, 5]. Importantly, miR-155 is one of many highly-expressed miRNAs in the inflamed colonic mucosa of both UC and CD patients [6, 7]. Moreover, miR-155 contributes to the early-life inflammatory stressors that trigger epithelial injury [8] and impair intestinal mucosa healing in IBD [9]. In spite of such known functions for miR-155, the mechanisms by which it impairs the intestinal barrier in IBD are poorly understood.

Profound hypoxia (or even anoxia) is present in the inflamed GI mucosa of IBD patients [10]. As a main regulator of cellular adaptive responses to hypoxia [11], hypoxia-inducible factor 1 (HIF-1), which is composed of HIF-1α and HIF-1β subunits, is also involved in many signaling pathways of tissue-protection and anti-inflammatory regulation [12, 13]. HIF-1α is degraded after being hydroxylated by prolyl hydroxylases (PHD), which lose this function under hypoxia [14]. Furthermore, HIF-1α is induced in the inflamed mucosa of both mouse models of colitis and IBD patients [15, 16] and can improve epithelial barrier function by regulating intestinal trefoil factor (TFF) as well as alleviate many kinds of intestinal inflammation [17–19]. Interestingly, by regulating the expression of HIF-1α, miR-155 promotes endothelial cell maturation and is involved in hypertrophic scar fibroblast formation [20, 21]. However, the underpinnings of HIF-1α regulation by miR-155 in IBD are not well understood.

Here, we hypothesize that miR-155 contributes to intestinal barrier dysfunction in dextran sulfate sodium (DSS)-induced mice colitis by regulating the HIF-1α/TFF-3 axis. In this study, we observed that miR-155 inhibition by antagomir treatment ameliorated DSS-induced colitis in mice and improved the expression of HIF-1α. We also found that intestinal mucosal barrier dysfunction and loss of TJ proteins in DSS-induced colitis is relieved by treatment with miR-155 antagomir or FG-4497. In addition, we revealed that the loss of function of the HIF-1α/TFF-3 axis in DSS-induced colitis in mice may promote intestinal barrier dysfunction.

## RESULTS

### MiR-155 antagomir reduced miR-155 levels in DSS-induced colitis

**Figure 1:**
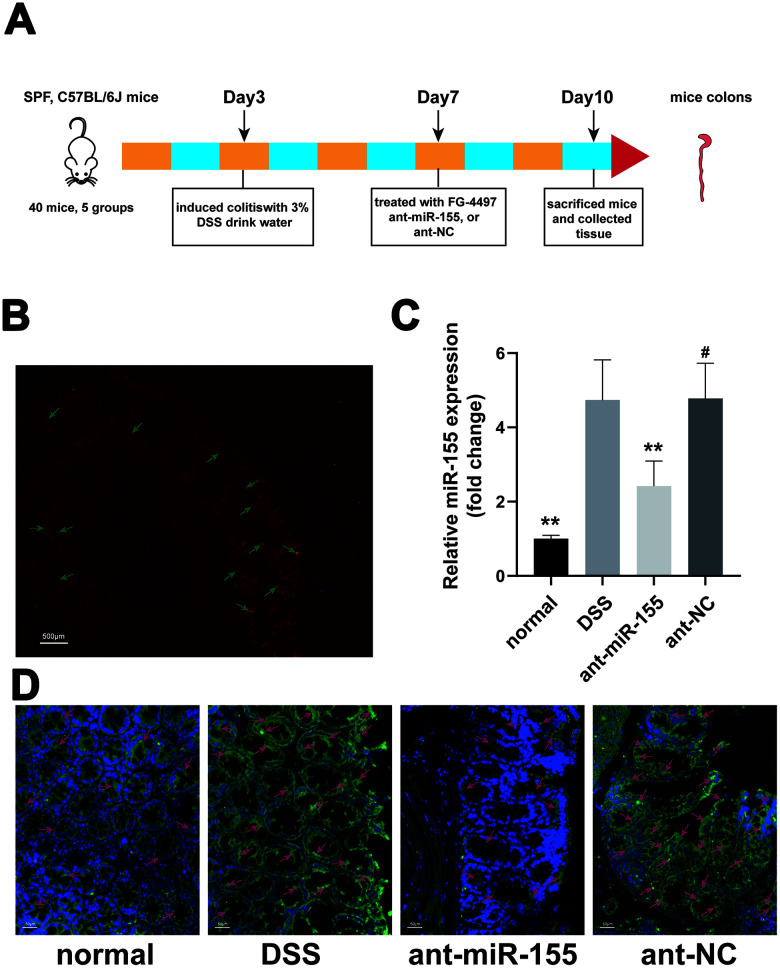
**MiR-155 antagomir reduced miR-155 level in DSS-induced colitis.** (**A**) The schema of the animal experiment. (**B**) Location of miR-155 antagomir in mouse colon. Red marked by green arrows was positive signal (magnification ×20). (**C**) QRT-PCR analysis of miR-155 level in mice colonic tissue. Each bar represents mean ± SD, n=8 from each group. ^#^P > 0.05, **P < 0.01 vs. DSS group. (**D**) In *situ* hybridization analysis of miR-155 in mice colon. Green marked by red arrows was positive signal for miR-155 (magnification ×200).

We tested the effects of intraperitoneal miR-155 antagomir injection into mice. Figure 1B shows that miR-155 antagomir was mainly located in epithelial and submucosal cells. Next, our q-PCR experiments showed that miR-155 expression was increased 4.74-fold in DSS-induced colon tissues compared with that in normal controls. However, miR-155 antagomir decreased miR-155 levels (P < 0.01) (Figure 1C). Furthermore, fluorescence *in situ* hybridization assays confirmed that miR-155 was characteristically distributed in colon epithelial cells and was expressed at higher levels in DSS colon tissues compared to normal controls, with miR-155 antagomir reducing miR-155 levels (P<0.01) (Figure 1D). Our results showed that miR-155 levels were increased in DSS-induced colitis, which is consistent with our previous results [22], and that miR-155 antagomir reduced miR-155 levels in mice colonic tissues.

### MiR-155 contributes to DSS-induced colitis in mice

**Figure 2:**
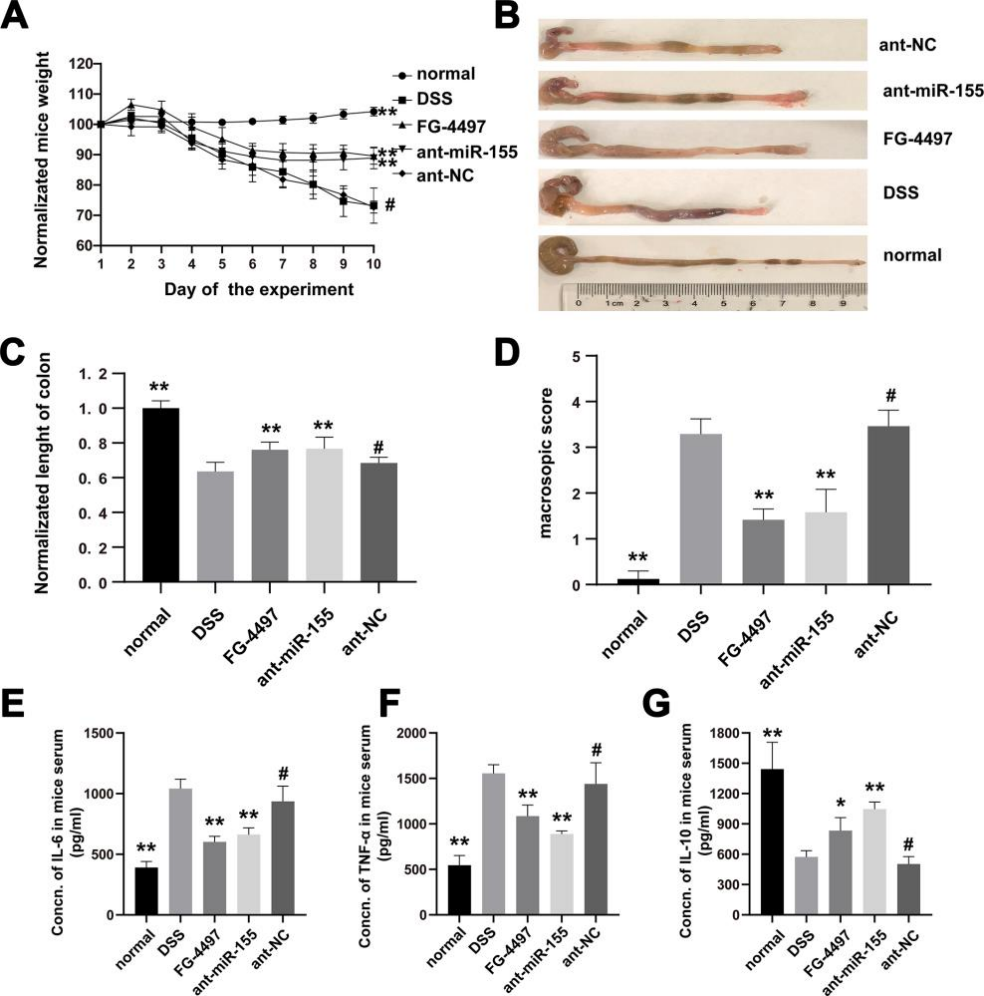
**MiR-155 contributed to DSS-induced colitis in mice.** (**A**) Normalized weights of mice were presented. (**B**) Colons gross appearances from each group were showed. (**C**) Colonic lengths were measured. (**D**) Macroscopic score of each group colonic tissue was presented. (**E**–**G**) IL-6, TNF-α, and IL-10 in mice serum were measured with ELISA. Each bar represents mean ± SD, n=8 from each group. ^#^P > 0.05, *P < 0.05, **P < 0.01 vs. DSS group.

We recorded weight, stool consistency, stool occult blood, and general physical symptoms daily for all groups of mice in our study. As showed in Figure 2A, mice treated with DSS had decreased weight compared with that of normal mice (P < 0.01). However, the weight loss was gradually decreased from day seven after administration of the miR-155 antagomir. Colon length was decreased by DSS (Figure 2B, 2C) as compared with that in normal group (P < 0.01) suggesting acute colonic inflammation in DSS mice. However, the average colonic length of the ant-miR-155 group was 1.21-fold that of the DSS group. In the ant-miR-155group, the damage of colonic mucosa was alleviated as indicated by the reduced macroscopic damage score, which was 76% of that in the DSS group (p<0.01) (Figure 2D). We used ELISA to measure the levels of inflammatory and anti-inflammatory cytokines in mice serum as a proxy for intestinal inflammation in mice from each group (Figure 2E–2G). Pro-inflammatory cytokines IL-6 and TNF-α were increased by 2.66 folds and 2.76 folds in the DSS group, respectively, compared to normal controls (P<0.01). However, the levels of these pro-inflammatory cytokines decreased after treatment with miR-155 antagomir (P<0.01). Interestingly, the levels of anti-inflammatory cytokine, IL-10, showed the opposite behavior. In addition, FG-inhibited the function of PHD and decreased the degradation of the HIF-1α protein. As showed in Figure 2, after treatment with FG-4497, the weight of mice stabilized and colon length shortening was less than that of the DSS group. Pro-inflammatory cytokine levels decreased while anti-inflammatory cytokine levels increased (p<0.01) in mice serum after FG-4497 treatment.

**Figure 3:**
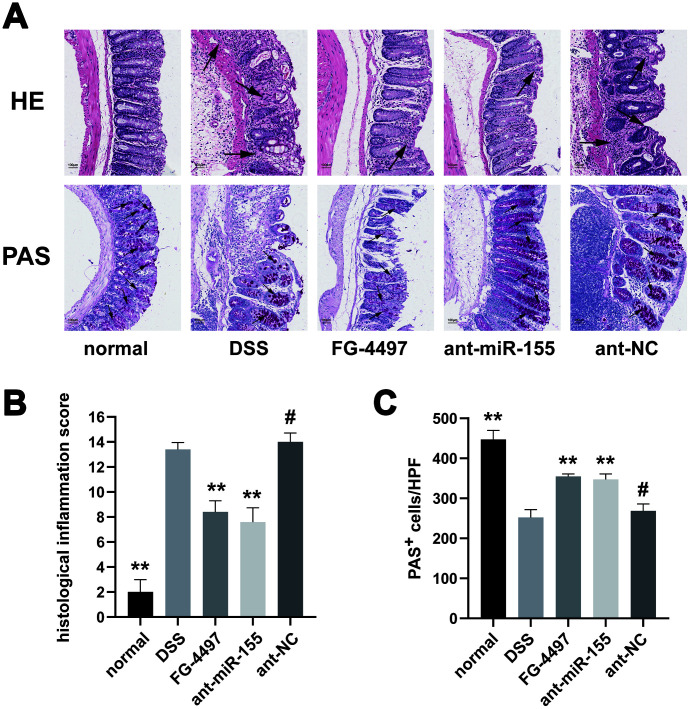
**Histopathological evaluation of intestinal inflammation in all groups.** (**A**) Hematoxylin and eosin (HE) and Periodic Acid-Schiff (PAS) analysis of colon specimens (magnification ×100). Massive inflammation cell infiltration, mucosal erosion, and submucosa edema were observed throughout the colons in the DSS and ant-NC group. For PAS stain, red particles marked by black arrows represented the positive change. (**B**) Histological inflammation scores in all groups were presented. (**C**) PAS^+^ cells per high power field in all groups were presented. Each bar represents mean ± SD, n=5 from each group. ^#^P > 0.05, *P < 0.05, **P < 0.01 vs. DSS group.

The pathological changes in mice colon were showed by HE and PAS stain (Figure 3). Mucosal erosion with mass inflammatory cells infiltration was observed throughout the colons of DSS mice with HE. On the other hand, miR-155 antagomir treatment alleviated such lesions. The histological inflammation scores were increased in DSS group compared to the normal group and were decreased by miR-155 antagomir or FG-4497 treatment (Figure 3B). Goblet cells were depleted in the DSS group compared to controls as measured by PAS but was rescued by miR-155 antagomir or FG-44497 treatment (Figure 3A, 3C). Our results show that miR-155 promotes DSS-induced colitis while miR-155 antagomir exerts therapeutic functions. These results are consistent with a previous report demonstrating that FG-4497 relieves TNBS-induced colitis [23].

### MiR-155 promotes intestinal barrier dysfunction in DSS-induced colitis

**Figure 4:**
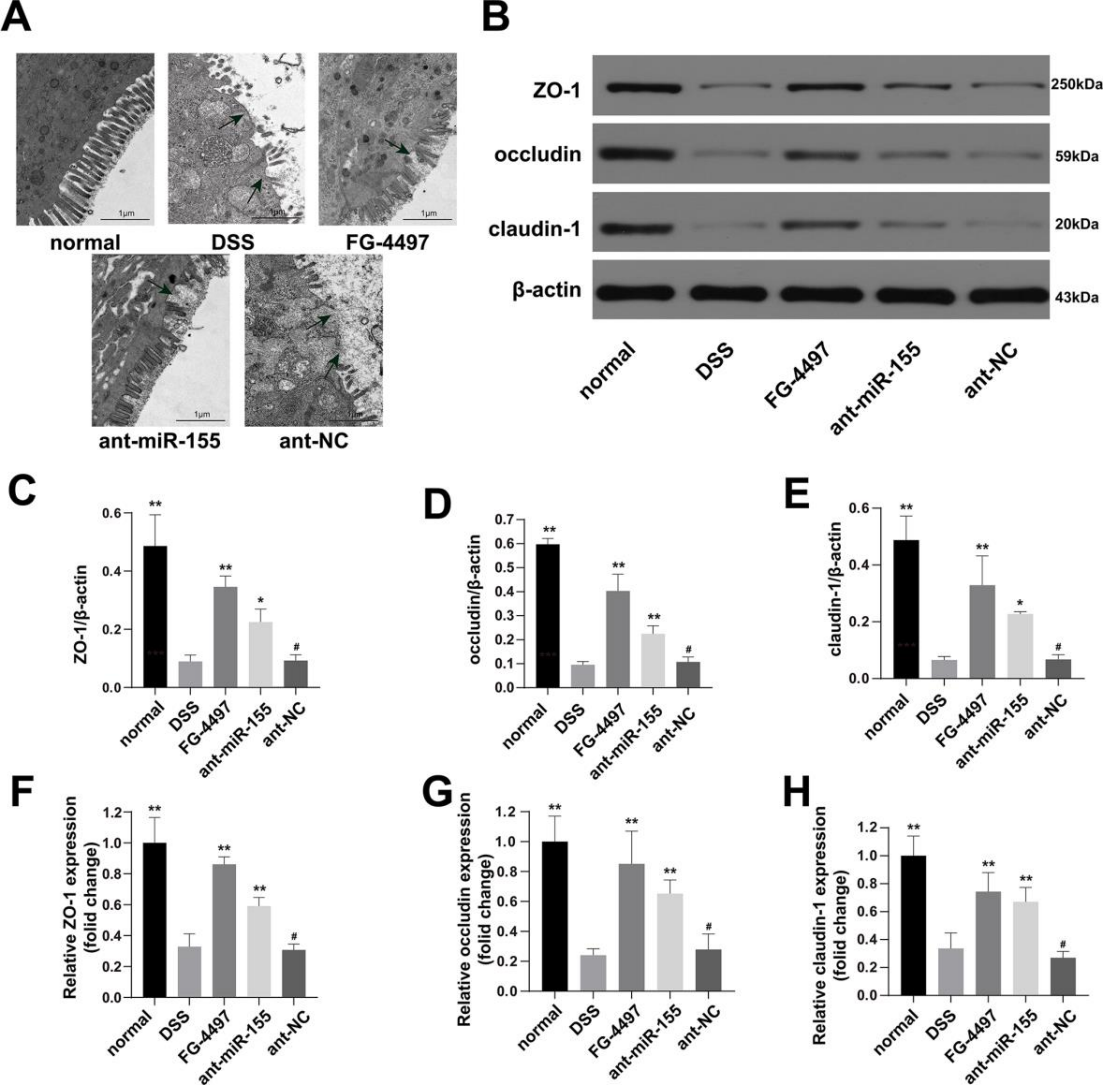
**MiR-155 antagomir ameliorated intestinal barrier dysfunction in DSS-induced colitis.** (**A**) Transmission electron microscopic (TEM) assay of colon specimens (magnification ×5000). The damages of epithelial cell villous and epithelial cells were significant in DSS and ant-NC group. (**B**) Western blotting analysis of TJ proteins (ZO-1, occludin, and claudin-1). (**C**–**E**) representative protein levels of ZO-1/β-actin, occludin/β-actin, and claudin-1/β-actin (n=3). (**F**–**H**) Relative mRNA of ZO-1, occludin, and claudin-1 in mice colonic tissue were measured by qPCR (n=5). Each bar represents mean ± SD, ^#^P > 0.05, *P < 0.05, **P < 0.01 vs. DSS group.

**Figure 5:**
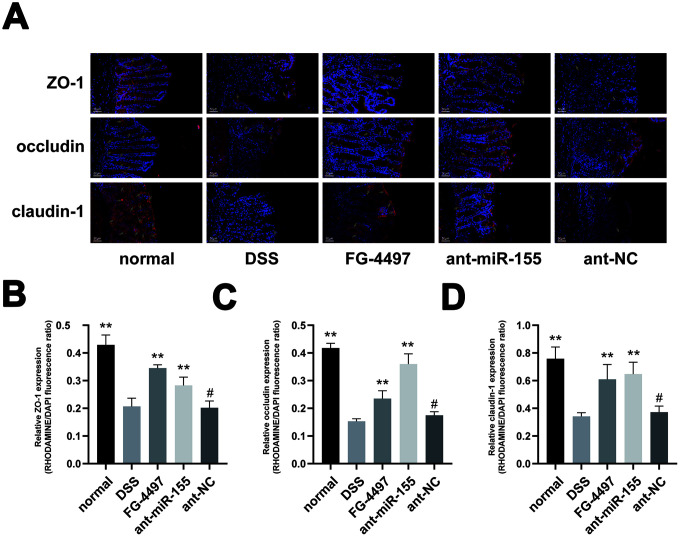
**Immunofluorescence assay presented the expression of TJ proteins in mice colon.** (**A**) Immunoflorescent staining of ZO-1, occludin, and claudin-1 in the colonic tissues. (magnification, ×200) Red signal (rhodamine) labeled by green arrows was positive for these proteins. DAPI (blue) was used to counterstain of nuclei. (**B**–**D**) Relative ZO-1, occludin, and claudin-1 expression measured by rhodamine and DAPI fluorescence ratio in all groups were presented. Each bar represents mean ± SD, n=5 from each group, ^#^P > 0.05, *P < 0.05, **P < 0.01 vs. DSS group.

To evaluate the dysfunction of intestinal barrier in DSS-induced colitis, we collected transmission electron microscopy (TEM) images of fixed mice colon tissues (Figure 4A). We observed a depletion of intestinal epithelial cell villus in the DSS group compared with the normal group, which was relieved by administration of miR-155 antagomir or FG-4497. Tight junction (TJ) proteins, mainly occludin, claudins and zonula occludens (ZO), help to maintain the intestinal barrier function. With western blot and immunofluorescence (Figure 4B–4E and Figure 5), we found that the protein levels of occludin, claudin-1, and ZO-1 were reduced in DSS-induced colitis colonic tissues, and the reduction in miR-155 levels by miR-155 antagomir treatment increased the expression of these proteins (P<0.01). We further confirmed this trend by measuring miR-155 mRNA using qPCR (Figure 4F–4H). FG-4497 treatment elicited similar results. This intestinal barrier protective function may result from increased expression of HIF-1α. Our results suggest that high expression of miR-155 in DSS-induced colitis promotes intestinal barrier dysfunction.

### MiR-155 downregulated the expression of HIF-1α in DSS-induced colitis mouse colonic tissues

**Figure 6:**
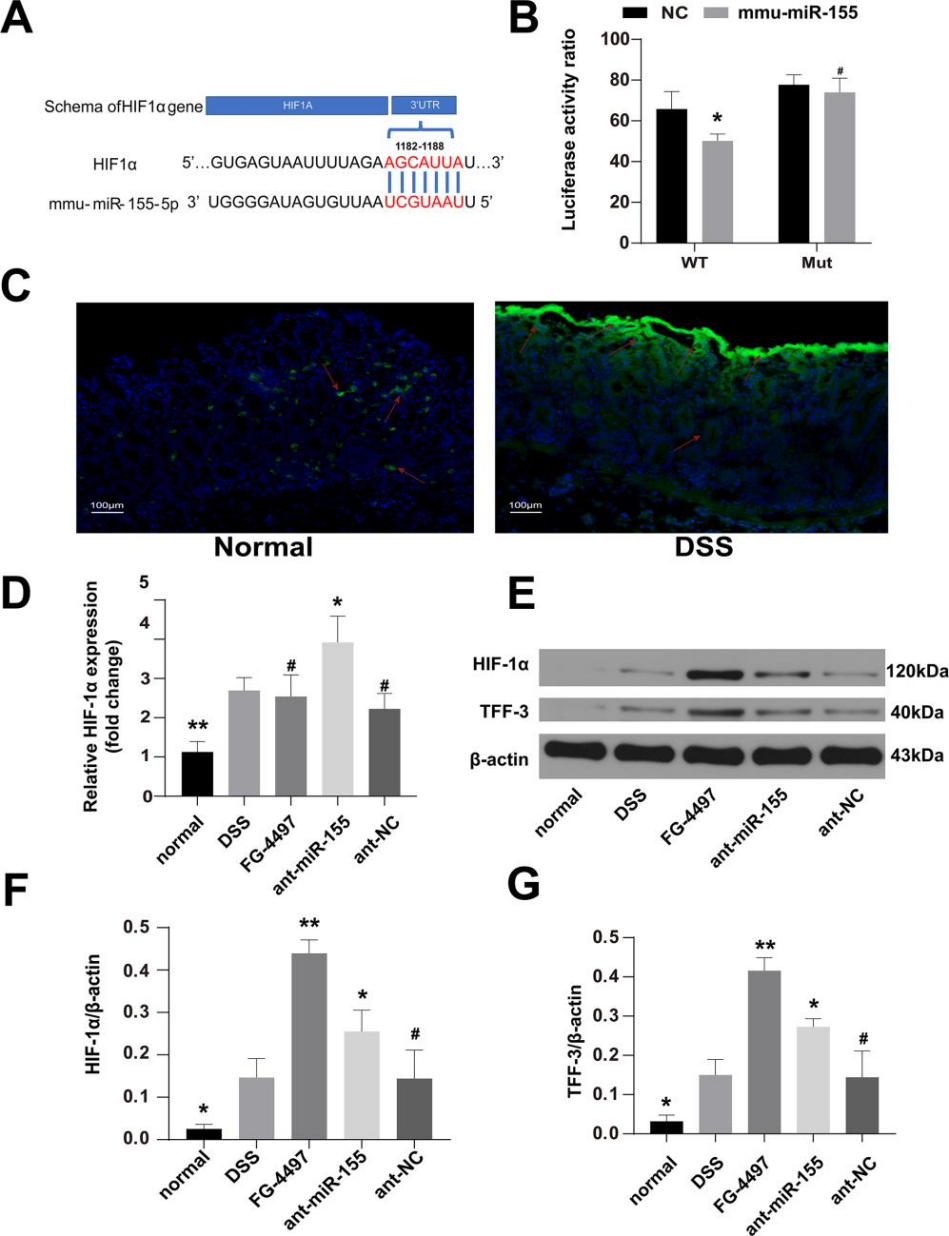
**MiR-155 dilapidated intestinal barrier may by targeting HIF-1α/TFF-3 axis.** (**A**) Wild-type sequences of HIF-1α for miR-155 target. (**B**) Dual luciferase report assay of HIF-1α 3’-UTR wild type or MUT along with miR-155. ^#^P > 0.05, *P < 0.05 vs. Negative Control. (**C**) The hypoxic station in normal and DSS-induced colitis mice colon labeled by hypoxia Probes. Green was the positive signal. (magnification ×100). (**D**) Relative mRNA of HIF-1α in mice colonic tissue were measured by qPCR (n=5). (**E**) Western blotting analysis of HIF-1α proteins and TFF-3 proteins in mice colon. (**F**, **G**) Representative protein levels of TFF-3/β-actin and HIF-1α/β-actin(n=3). Each bar represents mean ± SD. ^#^P > 0.05, *P < 0.05, **P < 0.01 vs. DSS group.

HIF-1α was predicted as one of the target genes of miR-155 with Target Scan and miRDB. To illuminate the regulation of HIF-1α by miR-155 in DSS-induced inflammation in mouse colonic tissues, we first conducted dual luciferase reporter assay in HEK293T cells. The results validated the direct interaction of miR-155 and HIF-1a (Figure 6A). The luciferase activity from the HEK293T cells transfected with wild type HIF-1α3’-UTR was decreased compared with that of HEK293T cells transfected with mutated HIF-1α3’-UTR and miR-155 (P<0.05) (Figure 6B). We also confirmed that hypoxia was aggravated in DSS-induced colitis mouse colonic tissues using hypoxia Probes (Figure 6C). We then quantified HIF-1α mRNA levels in these tissues using qPCR and found that they were elevated compared to those in normal controls and that miR-155 antagomir elevated them further (Figure 6D). We also measured HIF-1α protein expression in mouse colonic tissues using Western blotting and found that HIF-1α protein levels were increased by 1.87 folds in the presence of ant-miR-155 treatment compared to those in the DSS group (Figure 6E, 6F). As expected, HIF-1α expression was also increased in the FG-4497 treatment group compared with that in DSS group (Figure 6D, 6F, 6G).

In conclusion, miR-155 treatment decreased the expression of HIF-1α in DSS-induced colitis mouse colonic tissues while miR-155 antagomir or FG-4497 treatments rescued this reduction, eliciting intestinal barrier protective function.

### MiR-155 may target the HIF-1α/TFF-3 axis in DSS-induced mice colitis

TFF-3, which is closely related with the intestinal barrier and regulated by HIF-1α [17], modulates the expression of TJ proteins in intestinal tissue [24]. Thus, we also explored the relationship between HIF-1α and TFF-3 in DSS-induced colitis. Results from our Western blotting experiments showed that TFF-3 levels were increased in mice treated with DSS compared to normal controls, miR-155 antagomir or FG-4497 treatment further increased TFF-3 levels in the DSS group (Figure 6E, 6G). These results suggest that the target of miR-155 that contributes to intestinal barrier dysfunction in DSS-induced colitis may be the HIF-1α/TFF-3 axis.

## DISCUSSION

The intestinal epithelium acts not only a barrier that protects the sterile lamina propria from the anoxic and microorganism-rich lumen, but also a connection between the gut microbiota and the mucosal immune system [25, 26]. Numerous genetic studies highlight that many susceptibility genes of irritable bowel disorder (IBD) are involved in maintaining the integrity and normal functions of the intestinal barrier, such as mucus and glycoprotein regulation (MUC19) [27], MUC3 [28], epithelial differentiation (HNF4a) [29], and membrane transport (ITLN1) [30]. Dysfunction of the intestinal barrier is a hallmark of IBD [2]. Furthermore, defects or deficiencies in intestinal epithelial TJ proteins increase intestinal permeability and promote the development of intestinal inflammation [31]. Here, we showed that DSS treatment induces damage in the intestinal barrier of mouse colonic tissues and decreases the expression of intestinal epithelial TJ protein including ZO-1, occludin, and claudin-1. These data provide insight into the pathological mechanisms underlying IBD, which could open new treatment avenues for IBD.

HIF is known to exert protective functions on the intestinal barrier [17, 32, 33] and to interact with other regulators in the intestinal barrier protective pathway such as p-glycoprotein to clear xenobiotics [32]. Shao et al. [34] found that intestinal HIF-1α is essential to the adaptative response to alcohol-induced dysfunction of the intestinal barrier and influences the expression of TFF, claudin-1, and occludin. Furthermore, TFF-3 alleviated intestinal barrier dysfunction by reducing the expression of toll-like receptor 2 (TLR-2) [35] and TLR-4 [36]. Importantly, TFF-3 upregulated the expression of intestinal cell junctional complexes, thereby protecting the intestinal barrier [24]. Furthermore, prolyl hydroxylases inhibitor, dimethyloxalylglycine (DMOG), induces both HIF-1 and NF-kappaB activity in cultured intestinal epithelial cells, and is strongly reduces epithelial barrier dysfunction in DSS-induced colitis [18]. FG-4497-induced HIF-1α provides an overall beneficial influence on trinitrobenzenesulfonic acid (TNBS)-induced colitis, mainly because of its barrier protective functions [23]. Here, we showed that TFF-3 expression ameliorates intestinal barrier damage and that the expression of TJ proteins is increased by FG-4497-induced expression of HIF-1α. Our research results suggest that HIF-1α promotes the expression of TJ proteins with protective effects on the intestinal barrier possibly by upregulating TFF-3. The specific mechanisms by which HIF-1α regulates TFF-3 need to be further investigated, as well as the mechanisms by which elevated HIF-1α levels protect the intestinal barrier in mouse colonic tissues. MiR-155 promotes IBD and is increased in colonic tissues from IBD patients and animal models [6, 7, 22]. Furthermore, miR-155 contributes to increased gut permeability by suppressing the expression of E-Cadherin in “double-hit” colitis models [8] and impairs colonic healing by promoting the accumulation of double-strand breaks (DSBs) in injured colonic epithelium [9]. Our previous studies demonstrated that the levels of miR-155 are increased in different animal colitis models and promote an abnormal mucosal immune response [22, 37]. In agreement with such reports, our current study here shows that miR-155 levels were increased in DSS-induced colitis and that miR-155 antagomir reduces them, thereby relieving colitis. Furthermore, previous studies showed that miR-155 inhibits the expression of HIF-1α by binding the 3’ UTR of HIF-1a mRNA [20], which is consistent with our present research results. Here, we also found that the inhibition of miR-155 by miR-155 antagomir upregulated HIF-1α and was accompanied by a reduction in intestinal barrier damage and inflammation. Our results here suggest that miR-155 inhibits HIF-1α expression and thereby contributes to the intestinal barrier dysfunction in DSS-induced colitis, and highlights miR-155 antagomir and FG-4497 as potential therapeutic targets to treat human IBD.

## MATERIALS AND METHODS

### Animals and drugs

Male specific-pathogen free (SPF) C57BL/6J mice (7–8 weeks old) were provided by the Center for Disease Control of Hubei province and feed in SPF conditions in the experimental animal center of Huazhong University of Science and Technology (HUST, Wuhan, China). They were housed at room temperature (22–24 °C) and had free access to chow and water. In this study, all animal experiments were performed according to the Animal Research Institute Committee Guidelines of HUST and approved by the Institutional Animal Care and Use Committee of HUST. Mouse miR-155-5p antagomir and negative control antagomir were purchased from Gene Pharma Inc. (Shanghai, China). The sequence of miR-155-5p antagomir is 5'-ACCCCUAUCACAAUUAGCAUUAA-3'and the sequence of negative control antagomir is 5'-CAGUACUUUUGUGUAGUACAA-3'. Both are labeled with Cy3 at 5’. FG-4497 was synthesized at Fibro Gen, Inc. (San Francisco, CA). Hypoxyprobe™ Plus Kit was purchased from Hypoxyprobe, Inc.

### DSS-induced mice colitis and treatments

As showed in Figure 1A, after three days of adaptive feeding in the center, C57BL/6J mice were randomly divided into five groups: (1) normal control group (normal, n = 8); (2) DSS group (DSS, n = 8); (3) DSS+FG-4497 group (FG-4497, n = 8); (4) DSS+miR-155 antagomir group (ant-miR-155, n = 8); (5) DSS+miR-155 antagomir negative control group (ant-NC, n = 8). Colitis was induced in all mice groups except the wild-type group by adding 3.0% DSS (36–50 kDa; MP Biomedicals) in their drinking water for seven days. The mice in the FG-4497 group were injected intraperitoneally with 100 μl FG-4497 (dissolved in saline, 20mg/kg) daily for the last three days before tissue harvest, as previously described [23, 38]. At the same time, the mice in the ant-miR-155 and ant-NC groups were injected intraperitoneally with 100 μl of antagomiR-155 and negative control (dissolved in saline at 2 mg/ml), correspondingly. All the mice from other groups were injected intraperitoneally with 100 μl saline. The weights, stool consistency, and stool occult blood of all groups were recorded daily. On day 10, animals were sacrificed after collecting blood from their eyeballs, and colons were excised and prepared for histological analysis and other assays. Additionally, some mice from the normal and DSS groups were sacrificed one hour after intraperitoneal injection with Hypoxyprobe™-1(60 mg/kg body weight).

### Dual luciferase report assay

The wild type 3’UTRs and mutant 3’UTRs of HIF-1α was cloned downstream of the firefly luciferase gene in the pmirGLO vector. HEK293T cells were co-transfected with pmirGLO and miR-155 or a control miRNA with Lipofectamine 2000 (Invitrogen, Carlsbad, CA, USA) as previously described [39]. Luciferase activity was measured by Dual-Luciferase Reporter Assay System (Promega, E1910).

### *In situ* hybridization and immunofluorescence

Fluorescence *in situ* hybridization assay was used to observe the location and expression of miR-155-5p in mouse colon. As previously described [40], paraffin slices of mouse colon were hybridized with FITC-labeled miR-155-5p probes. The mucosal barrier related TJ proteins including ZO-1, occludin, and claudin-1 were assessed in mouse colon by immunofluorescence laser scanning confocal microscopy (FV3000; Olympus, Japan).

### Hematoxylin and eosin (HE) and periodic acid-Schiff (PAS)

Mouse colon tissues fixed in neutral-buffered formalin were embedded in paraffin. Then they were serially sectioned using a microtome (2μm). The tissue sections were stained with hematoxylin and eosin (H&E) for histological analysis and stained with PAS for mucous-containing goblet cells as described previously [41]. All histological images were taken with an optical microscope (E100; Nikon, Japan). For the histological inflammation score evaluated from H&E staining, three sections per mouse were blindly scored by two researchers. Five aspects including epithelial cell erosion, goblet cell depletion, architecture of the bowel, and infiltration of neutrophils and mononuclear cells, were evaluated with a maximum score of 15. Goblet cells were quantified in PAS stained sections (10 high power fields/section).

### Electron microscopy

Transmission electron microscopy (TEM) imaging was performed as previous described [42]. The mouse colon specimens were embedded in epoxy resin and cut into ultrathin sections (60–70 nm). Stained sections were visualized using a Hitachi-HT7700 electron microscope (Tokyo, Japan).

### RT-PCR for miRNA and mRNA

The levels of miR-155 and mRNA of HIF-1α, ZO-1, occludin, and claudin-1 in mouse colon tissues were measured with quantitative RT-PCR as previously described [22]. β-actin and U6 RNA were used as internal controls for mRNA and miRNA. The primers of all miRNA and mRNAs were: 5’-CTCAACTGGTGTCGTGGAGTCGGCAATTCAGTTGAGACCCCTAT-3’, 5’-ACACTCCAGCTGGGTTAATGCTAATCGTGAT-3’, and 5’- TGGTGTCGTGGAGTCG-3’ for miR-155-5p; 5’-AACCCTCGAGGCGTTTCCTAATCTCATT-3’and 5’-AACCGCGGCCGCAAGCTGGAAGGTTTGTG-3’ for HIF-1α mRNA,5’-TACCTCTTGAGCCTTGAACTT-3’ and 5’-CGTGCTGATGTGCCATAATA-3’ for ZO-1 mRNA, 5’-GGCTTCTCTGGGATGGATCG-3’ and 5’-TTTGCGAAACGCAGGACATC-3’ for claudin-1 mRNA, 5’-CTTCTGCTTCATCGCTTCC-3’ and 5’-CTTGCCCTTTCCTGCTTTC-3’ for claudin mRNA, 5’-AACGCTTCACGAATTTGCGT-3’ and 5’-CTCGCTTCGGCAGCACA-3’ for U6, 5’-CATCCGTAAAGACCTCTATGCCAAC-3’ and 5’-ATGGAGCCACCGATCCACA-3’ for β-actin mRNA.

### Western blot

Western blot was performed to quantify HIF-1α, TFF-3, ZO-1, occludin, and claudin-1 protein levels in mouse colonic tissues as previously described [43]. Anti-activated HIF-1α antibody (CST# 3716S), anti-activated TFF-3 antibody (MBS2001572), anti-activated ZO-1 antibody (Catalog # 61-7300), anti-activated occludin antibody (Catalog # 71-1500), and anti-activated claudin-1 antibody (Catalog # 51-9000) were used to Western blot as primary antibodies.

### Enzyme-linked immunosorbent assay(ELISA)

The levels of IL-6, IL-10, and TNF-αin in mouse serum were measured using ELISA kits (Bioswamp, Wuhan, China) according to the manufacturer's instructions. The assays of these cytokines employ the quantitative sandwich enzyme immunoassay technique and absorbance was measured at 450 nm using a microplate reader.

### Statistical analysis

Data were recorded as mean ± standard deviation (SD). Two groups were analyzed with unpaired two tailed Student *t*-test; datasets including data from more than two groups were analyzed with One-way ANOVA with Bonferroni *post hoc* test. P < 0.05 was considered statistically significant. SPSS 22.0 was used for data analysis.
